# An exponentiated XLindley distribution with properties, inference and applications

**DOI:** 10.1016/j.heliyon.2024.e25472

**Published:** 2024-01-29

**Authors:** Abdullah M. Alomair, Mukhtar Ahmed, Saadia Tariq, Muhammad Ahsan-ul-Haq, Junaid Talib

**Affiliations:** a Department of Quantitative Methods, School of Business, King Faisal University, 31982, Al-Ahsa, Saudi Arabia; b School of Statistics, Minhaj University Lahore, Lahore, Pakistan; c College of Statistical Sciences, University of the Punjab, Lahore, Pakistan

**Keywords:** XLindley distribution, Generalization, Reliability measures, Inference, Bayesian, Data analysis

## Abstract

In this paper, we propose exponentiated XLindley (EXL) distribution. The novel model is adaptable due to the mixt shapes of its density and failure rate functions. The following key statistical properties of EXL distribution are derived: quantile function, moments, hazard function, mean residual life, and Rényi entropy. The parameters are estimated using the maximum likelihood, Anderson Darling, Cramer von Misses, maximum product spacing, ordinary and weighted least square estimation procedures. To examine the behavior of the estimate, Monte Carlo simulation is used. Further Bayesian technique is also utilized to estimate the EXL parameters. The traceplot and Geweke diagnostics are used to track the convergence of simulated processes. The applicability of the EXL distribution is demonstrated by three datasets from different domains such as mortality rate due to COVID-19, precipitation in inches, and failure time for repairable items. The proposed distribution provides efficient results as compared to renowned competitive distributions.

## 1 Introduction

Modeling of lifetime data remained a matter of attraction for statisticians to deal with probabilistic reasoning. Lifetime models play an imperative role in fields like engineering, management, biological and health sciences, etc. For the depiction and projection of real-world phenomena, numerous probability models have been devised and utilized for the mentioned purpose. There is always some space to develop new models that are more flexible or have better fitting in special cases related to real life. This goal can be achieved using various generalization approaches such as transmuted approach [1], exponentiated-G [2], Beta-G [3], Weibull-G [4], Alpha power transformed [5], odd Fréchet-G [6], truncated Burr X-G [7], and Teissier-G [8] among others.

The exponentiated family, a very flexible approach for the addition of new a parameter to a continuous distribution, was originally introduced by Ref. [2]. It was used to generalize exponential, gamma, Weibull, and Pareto distribution and according to the results, it is evident that this approach enhances the adaptability of the model. Some examples of generalized distributions using this technique; are exponentiated Fréchet [9], exponentiated Gumble [10], exponentiated gamma [11], exponentiated Pareto [12], exponentiated Weibull [13], exponentiated Lomax [14], exponentiated power Lindley [15], exponentiated Lindley geometric [16], and exponentiated discrete Lindley [17].

Lindley distribution is introduced by Ref. [18] in the context of fiducial and Bayesian inference. It is also used for reliability analysis. Lindley distribution is the combination of two probability (gamma and exponential) models. The probability density function (pdf) is

$$f\left(z;\theta \right)=\frac{\delta^{2}}{{\left(1+\delta \right)}^{2}}\left(1+z\right)e^{-\delta z},\delta >0,z>0$$

One of the flexible and simplest lifetime models was recently introduced by Ref. [19]. It was termed “XLindley distribution” after being derived as a finite combination of exponential and Lindley models. A thorough examination of the salient features of the derived distribution indicated that the XLindley distribution is a more effective model than Lindley and provides a greater basis for real-world applications. The pdf and cumulative distribution function (cdf) of the XLindley distribution are shown.

$$f\left(z\right)=\frac{\delta^{2}\left(2+\delta +z\right)}{{\left(1+\delta \right)}^{2}}e^{-\delta z},\delta >0,z>0$$

and

$$F\left(z\right)=1-\left(1+\frac{\delta z}{{\left(1+\delta \right)}^{2}}\right)e^{-\delta z}\text{.}$$

Some authors further introduced some extended forms of XLindley distribution such as Unit-XLindley distribution [20], Poisson XLindley distribution [21], Power XLindley distribution [22], Quasi-XLindley distribution [23], and new discrete XLindley distribution [24].

The major goal for the work was the development of a novel two-parameter flexible lifetime distribution. This work has the following key goals.

• To provide a new generalization of the XLindley distribution utilizing the exponentiated parameter induction technique. The new model is named “Exponentiated XLindley distribution”. The new model has configurable density and hazard functions that can be used to model different types of datasets.
• To derive and investigate some of its most important mathematical and reliability aspects.
• To estimate the model parameters using various classical and robust estimation techniques. Bayesian approach is also used.
• The proposed distribution is used to analyze three datasets from different areas.

The rest of the study is systematized as follows: Section 2 is dedicated to the derivation of the new model and its shape analysis. Section 3 derives several key statistical features. Point parameters estimation is achieved in Section 4. Section 5 assesses the selection of an effective estimating technique using a comprehensive simulation study. Three examples from different fields are given in Section 6 to prove the flexibility of the EXL distribution. Bayesian analysis is conferred in Section 7. Some concluding remarks are given in Section 8.

## 2 Derivation of new distribution and its shape analysis

A generalized form of XLindley distribution is proposed using power to cdf transformation $G\left(X\right)={\left[F\left(Z\right)\right]}^{\alpha }$, where Z is the random variable that follows the XLindley distribution with parameter δ. A random variable X follows exponentiated XLindley (EXL) model, symbolically it is written as X∼EXL(α,δ). The cdf of EXL is as follows:

(1) $$F\left(x\right)={\left[1-\left(1+\frac{\delta x}{{\left(1+\delta \right)}^{2}}\right)e^{-\delta x}\right]}^{\alpha }\;\alpha ,\delta \geq 0,x\geq 0\text{,}$$

where δ is the scale and α is the shape parameters of EXL distribution.

The pdf of EXL corresponding to equation (1) is as follows:

(2) $$f\left(x\right)=\frac{\alpha \delta^{2}\left(2+\delta +x\right)e^{-\delta x}}{{\left(1+\delta \right)}^{2}}{\left[1-\left(1+\frac{\delta x}{{\left(1+\delta \right)}^{2}}\right)e^{-\delta x}\right]}^{\alpha -1}\text{,}$$

The alternative form of pdf is given in equation (3)

(3) $$g\left(x\right)=\sum_{k=0}^{\infty }\sum_{s=0}^{\infty }\frac{\alpha \delta^{s+2}{\left(-1\right)}^{k}\left(\begin{array}{c} \alpha -1\\ k \end{array}\right)\left(\begin{array}{c} k\\ s \end{array}\right)}{{\left(1+\delta \right)}^{2s+2}}\left(\left(2+\delta \right)x^{s}+x^{s+1}\right)e^{-\left(k+1\right)\delta x}\text{.}$$

Furthermore, survival and hazard function (HF) of EXL distribution are

$$S\left(x\right)=1-{\left[1-\left(1+\frac{\delta x}{{\left(1+\delta \right)}^{2}}\right)e^{-\delta x}\right]}^{\alpha }\text{,}$$

and

$$h\left(x\right)=\frac{\frac{\alpha \delta^{2}\left(2+\delta +x\right)e^{-\delta x}}{{\left(1+\delta \right)}^{2}}{\left[1-\left(1+\frac{\delta x}{{\left(1+\delta \right)}^{2}}\right)e^{-\delta x}\right]}^{\alpha -1}}{1-{\left[1-\left(1+\frac{\delta x}{{\left(1+\delta \right)}^{2}}\right)e^{-\delta x}\right]}^{\alpha }}\text{.}$$

### 2.1 Limiting behavior of density function and HF

In this section limiting demonstration of density and the HF of EXL distribution are discussed. The behavior of the pdf at the lower limit (x→0) is given below

$$\lim_{x\to 0}f\left(x\right)=\left\{ \begin{array}{cc} \infty & \;for\;0<\alpha <1\\ \frac{\delta^{2}\left(2+\delta \right)}{{\left(1+\delta \right)}^{2}} & for\;\alpha =1\\ 0 & for\;\alpha >1 \end{array}\right.$$

**Fig. 1:**
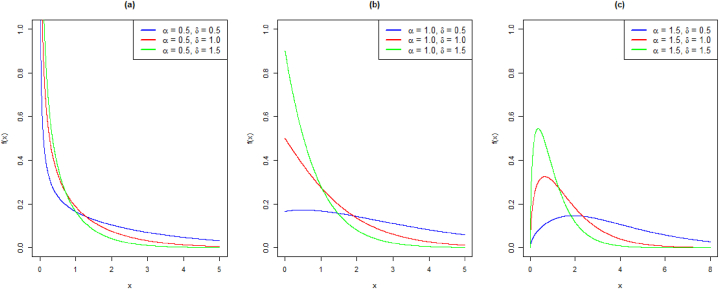
Density plots of EXL distribution: (a) α = 0.5, (b) α = 1.0, (c) α = 1.5 and varying values of δ

The density visualizations for different parameter choices are shown in Fig. 1 (a)-(c).

The limiting presentation of the HF at the lower and upper limit of variable x is given below:

$$\lim_{x\to 0}h\left(x\right)=\left\{ \begin{array}{cc} \infty & \;for\;0<\alpha <1\\ \frac{\delta^{2}\left(2+\delta \right)}{{\left(1+\delta \right)}^{2}} & for\;\alpha =1\\ 0 & for\;\alpha >1 \end{array}\right.$$

and

$$\lim_{x\to \infty }h\left(x\right)=\left\{ \begin{array}{c} \infty 0<\alpha <\infty \&\;\alpha \neq 1\\ \delta \;\alpha =1\; \end{array}\right.$$

**Fig. 2:**
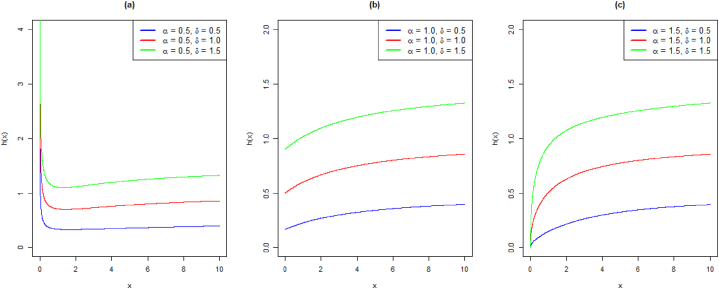
Hazard function curves of EXL distribution: (a) α = 0.5, (b) α = 1.0, (c) α = 1.5 and varying values of δ

It follows that the parameter α causes various shapes of the pdf and HF. To demonstrate the previously mentioned prerequisites, we plot the HF for various parameter choices in Fig. 2 (a)-(c).

## 3 Statistical properties of EXL distribution

This section contains the mathematical derivation of several important characteristics such as linear presentation, mode, quantile function, moments, and order statistics, as well as entropies.

### 3.1 Mode of the EXL distribution

Taking the log of equation (4), differentiating for x, and equating it to zero, we get the following expression

$$\frac{1}{2+\delta +x}-\delta +\frac{\delta \left(\alpha -1\right)\left(1+\delta x\right)e^{-\delta x}}{{\left(1+\delta \right)}^{2}\left[1-\left(1+\frac{\delta x}{{\left(1+\delta \right)}^{2}}\right)e^{-\delta x}\right]}=0$$

The exact solution of mode is not tractable, so the values of mode can be derived numerically by computing the above equation.

### 3.2 Quantile function

The quantile function for the EXL model is

$$x_{u}=-\frac{{\left(1+\delta \right)}^{2}}{\delta }-\frac{1}{\delta }W_{-1}\left[\frac{{\left(1+\delta \right)}^{2}}{e^{{\left(1+\delta \right)}^{2}}}\left(u^{\frac{1}{\alpha }}-1\right)\right]\text{.}$$

The proof is given in the Appendix.

You can calculate the median of EXL distribution by setting $u=\frac{1}{2}$, that is

$$x_{\frac{1}{2}}=-\frac{{\left(1+\delta \right)}^{2}}{\delta }-\frac{1}{\delta }W_{-1}\left[\frac{{\left(1+\delta \right)}^{2}}{e^{{\left(1+\delta \right)}^{2}}}\left({\left(\frac{1}{2}\right)}^{\frac{1}{\alpha }}-1\right)\right]\text{.}$$

### 3.3 Moments of EXL distribution

The moments of EXL distribution are

$$\mu_{r}^{\prime }=\sum_{k=0}^{\infty }\sum_{s=0}^{\infty }\frac{\alpha {\left(-1\right)}^{k}\left(\begin{array}{c} \alpha -1\\ k \end{array}\right)\left(\begin{array}{c} k\\ s \end{array}\right)}{{\left(1+\delta \right)}^{2s+2}}\frac{\left(r+s\right)!\left(\delta \left(2+\delta \right)\left(k+1\right)+\left(r+s+1\right)\right)}{{\left(k+1\right)}^{r+s+2}\delta^{r}}\text{.}$$

Proof: The ordinary moments are demarcated as $\mu_{r}^{\prime }=E\left(X^{r}\right)=\int_{-\infty }^{+\infty }x^{r}g\left(x\right)dx$. Now after replacing the pdf given in equation (3) yields

$$\mu_{r}^{\prime }=\sum_{k=0}^{\infty }\sum_{s=0}^{\infty }\frac{\alpha \delta^{s+2}{\left(-1\right)}^{k}\left(\begin{array}{c} \alpha -1\\ k \end{array}\right)\left(\begin{array}{c} k\\ s \end{array}\right)}{{\left(1+\delta \right)}^{2s+2}}\int_{0}^{\infty }x^{r}\left(\left(2+\delta \right)x^{s}+x^{s+1}\right)e^{-\left(k+1\right)\delta x}dx\text{.}$$

Letting $\left(k+1\right)\delta x=y\;and\;x=\frac{y}{\left(k+1\right)\delta }$.

$$\mu_{r}^{\prime }=\sum_{k=0}^{\infty }\sum_{s=0}^{\infty }\frac{\alpha \delta^{s+2}{\left(-1\right)}^{k}\left(\begin{array}{c} \alpha -1\\ k \end{array}\right)\left(\begin{array}{c} k\\ s \end{array}\right)}{{\left(1+\delta \right)}^{2s+2}}\left\{\frac{1}{{\left(k+1\right)}^{r+s+1}\delta^{r+s+1}}\left[\left(2+\delta \right)\int_{0}^{\infty }y^{r+s}e^{-y}dy+\frac{1}{\left(k+1\right)\delta }\int_{0}^{\infty }y^{r+s+1}e^{-y}dy\right]\right\}$$

$by\;using\;\int_{0}^{+\infty }z^{k-1}e^{-z}dz=\left(k-1\right)!$ we get

$$\mu_{r}^{\prime }=\sum_{k=0}^{\infty }\sum_{s=0}^{\infty }\frac{\alpha \delta^{s+2}{\left(-1\right)}^{k}\left(\begin{array}{c} \alpha -1\\ k \end{array}\right)\left(\begin{array}{c} k\\ s \end{array}\right)}{{\left(1+\delta \right)}^{2s+2}}\left[\frac{\left(r+s\right)!\left(\delta \left(2+\delta \right)\left(k+1\right)+\left(r+s+1\right)\right)}{{\left(k+1\right)}^{r+s+2}\delta^{r+s+2}}\right]$$

$$\mu_{r}^{\prime }=\sum_{k=0}^{\infty }\sum_{s=0}^{\infty }\frac{\alpha {\left(-1\right)}^{k}\left(\begin{array}{c} \alpha -1\\ k \end{array}\right)\left(\begin{array}{c} k\\ s \end{array}\right)}{{\left(1+\delta \right)}^{2s+2}}\frac{\left(r+s\right)!\left[\delta \left(2+\delta \right)\left(k+1\right)+\left(r+s+1\right)\right]}{{\left(k+1\right)}^{r+s+2}\delta^{r}}\text{.}$$

The mean of the EXL distribution is given under

$$E\left(X\right)=\sum_{k=0}^{\infty }\sum_{s=0}^{\infty }\frac{\alpha {\left(-1\right)}^{k}\left(\begin{array}{c} \alpha -1\\ k \end{array}\right)\left(\begin{array}{c} k\\ s \end{array}\right)}{{\left(1+\delta \right)}^{2s+2}}\frac{\left(1+s\right)!\left[\delta \left(2+\delta \right)\left(k+1\right)+\left(s+2\right)\right]}{{\left(k+1\right)}^{s+3}\delta }\text{.}$$

The variance of the EXL distribution is given

$$Var\left(X\right)=\mu_{2}=\mu_{2}^{\prime }-{\left(\mu_{1}^{\prime }\right)}^{2}$$

$$\mu_{2}=\sum_{k=0}^{\infty }\sum_{s=0}^{\infty }\frac{\alpha {\left(-1\right)}^{k}\left(\begin{array}{c} \alpha -1\\ k \end{array}\right)\left(\begin{array}{c} k\\ s \end{array}\right)}{{\left(1+\delta \right)}^{2s+2}}\frac{\left(2+s\right)!\left[\delta \left(2+\delta \right)\left(k+1\right)+\left(s+3\right)\right]}{{\left(k+1\right)}^{s+4}\delta^{2}}-{\left(\sum_{k=0}^{\infty }\sum_{s=0}^{\infty }\frac{\alpha {\left(-1\right)}^{k}\left(\begin{array}{c} \alpha -1\\ k \end{array}\right)\left(\begin{array}{c} k\\ s \end{array}\right)}{{\left(1+\delta \right)}^{2s+2}}\frac{\left(1+s\right)!\left[\delta \left(2+\delta \right)\left(k+1\right)+\left(s+2\right)\right]}{{\left(k+1\right)}^{s+3}\delta }\right)}^{2}$$

The skewness and kurtosis of the random variable X which follows EXL distribution can be obtained using the moment ratio

$$\gamma_{1}=\frac{\mu_{3}^{\prime }-3\mu_{2}^{\prime }\mu_{1}^{\prime }+2{\left(\mu_{1}^{\prime }\right)}^{3}}{{\left(\mu_{2}^{\prime }-{\left(\mu_{1}^{\prime }\right)}^{2}\right)}^{\frac{3}{2}}}$$

and

$$\gamma_{2}=\frac{\mu_{4}^{\prime }-4\mu_{3}^{\prime }\mu_{1}^{\prime }+6{\mu_{2}^{\prime }\left(\mu_{1}^{\prime }\right)}^{2}-3{\left(\mu_{1}^{\prime }\right)}^{4}}{{\left(\mu_{2}^{\prime }-{\left(\mu_{1}^{\prime }\right)}^{2}\right)}^{2}}$$

Table 1 depicts the behavior regarding mean, variance, skewness ($\gamma_{1}$), and kurtosis ($\gamma_{2}$) for Exponentiated XLindley distribution. It is evident from the table that skewness and kurtosis of EXL decreases for higher values parameters.

**Table 1:** Some computational statistics of EXL model for various parameters values.

δ	α	Mean	Variance	$\gamma_{1}$	$\gamma_{2}$
0.25	0.20	2.02	13.58	3.11	16.16
	0.50	4.17	23.37	2.01	8.63
	1.00	6.56	29.93	1.53	6.4
	2.00	9.43	33.83	1.26	5.5
	5.00	13.59	37.12	0.74	5.99
0.50	0.20	0.86	2.84	3.41	19
	0.50	1.81	5.08	2.21	9.82
	1.00	2.89	6.77	1.66	6.99
	2.00	4.23	7.92	1.34	5.79
	5.00	6.24	8.55	1.15	5.23
1.00	0.20	0.36	0.57	3.73	22.37
	0.50	0.77	1.04	2.43	11.37
	1.00	1.25	1.44	1.83	7.89
	2.00	1.86	1.75	1.47	6.31
	5.00	2.79	1.96	1.22	5.51
2.00	0.20	0.16	0.12	3.92	24.77
	0.50	0.34	0.22	2.58	12.55
	1.00	0.56	0.30	1.95	8.64
	2.00	0.83	0.38	1.56	6.81
	5.00	1.26	0.43	1.30	5.82
4.00	0.20	0.07	0.03	3.98	25.62
	0.50	0.16	0.05	2.62	12.99
	1.00	0.26	0.07	1.99	8.94
	2.00	0.39	0.08	1.60	7.03
	5.00	0.59	0.10	1.33	5.99

### 3.4 Rényi entropy

The Rényi entropy can be derived as

(4) $$I_{R}\left(\gamma \right)=\frac{1}{1-\gamma }\log\left(\int_{0}^{\infty }g^{\gamma }\left(x\right)dx\right)\;\gamma >0,\gamma \neq 1\text{.}$$

Considering the integral part

$$\int_{0}^{\infty }g^{\gamma }\left(x\right)dx=\int_{0}^{\infty }{\left[\frac{\alpha \delta^{2}\left(2+\delta +x\right)e^{-\delta x}}{{\left(1+\delta \right)}^{2}}{\left[1-\left(1+\frac{\delta x}{{\left(1+\delta \right)}^{2}}\right)e^{-\delta x}\right]}^{\alpha -1}\right]}^{\gamma }dx$$

Using the binomial expansions,

(5) $$g^{\gamma }\left(x\right)=\frac{\alpha^{\gamma }\delta^{2\gamma }{\left(2+\delta \right)}^{\gamma }{\left(1+\frac{x}{2+\delta }\right)}^{\gamma }}{{\left(1+\delta \right)}^{2\gamma }}e^{-\delta \gamma x}{\left[1-\left(1+\frac{\delta x}{{\left(1+\delta \right)}^{2}}\right)e^{-\delta x}\right]}^{\gamma \left(\alpha -1\right)}$$

Using the following binomial expansions.

i. ${\left(1+\frac{x}{2+\delta }\right)}^{\gamma }=\sum_{m=0}^{\infty }\left(\begin{array}{c} \gamma \\ m \end{array}\right)\frac{x^{m}}{{\left(2+\delta \right)}^{m}}$
ii. ${\left(1+\frac{\delta x}{{\left(1+\delta \right)}^{2}}\right)}^{k}=\sum_{s=0}^{\infty }\left(\begin{array}{c} k\\ s \end{array}\right){\left(\frac{\delta x}{{\left(1+\delta \right)}^{2}}\right)}^{s}$

The last part of equation (5) will be

$${\left[1-\left(1+\frac{\delta x}{{\left(1+\delta \right)}^{2}}\right)e^{-\delta x}\right]}^{\gamma \left(\alpha -1\right)}=\sum_{k=0}^{\infty }\sum_{s=0}^{\infty }\;{\left(-1\right)}^{k}\left(\begin{array}{c} \gamma \alpha -\gamma \\ k \end{array}\right)\left(\begin{array}{c} k\\ s \end{array}\right)\frac{\delta^{s}}{{\left(1+\delta \right)}^{2s}}x^{s}e^{-k\delta x}$$

Put this in equation (4)

$$g^{\gamma }\left(x\right)=\frac{\alpha^{\gamma }\delta^{2\gamma }{\left(2+\delta \right)}^{\gamma }}{{\left(1+\delta \right)}^{2\gamma }}e^{-\delta \gamma x}\sum_{m=0}^{\infty }\left(\begin{array}{c} \gamma \\ m \end{array}\right)\frac{x^{m}}{{\left(2+\delta \right)}^{m}}\sum_{k=0}^{\infty }\sum_{s=0}^{\infty }\;{\left(-1\right)}^{k}\left(\begin{array}{c} \gamma \alpha -\gamma \\ k \end{array}\right)\left(\begin{array}{c} k\\ s \end{array}\right)\frac{\delta^{s}}{{\left(1+\delta \right)}^{2s}}x^{s}e^{-k\delta x}$$

and

$$g^{\gamma }\left(x\right)=\sum_{m=0}^{\infty }\sum_{k=0}^{\infty }\sum_{s=0}^{\infty }\;\left(\begin{array}{c} \gamma \\ m \end{array}\right)\left(\begin{array}{c} \gamma \alpha -\gamma \\ k \end{array}\right)\left(\begin{array}{c} k\\ s \end{array}\right){\left(-1\right)}^{k}\frac{\alpha^{\gamma }\delta^{2\gamma +s}{\left(2+\delta \right)}^{\gamma -m}}{{\left(1+\delta \right)}^{2\gamma +2s}}x^{m+s}e^{-\left(k+\gamma \right)\delta x}\text{,}$$

$$g^{\gamma }\left(x\right)=\sum_{m=0}^{\infty }\sum_{k=0}^{\infty }\sum_{s=0}^{\infty }W_{m,k,s}\;x^{m+s}e^{-\left(k+\gamma \right)\delta x}$$

where $W_{mks}=\left(\begin{array}{c} \gamma \\ m \end{array}\right)\left(\begin{array}{c} \gamma \alpha -\gamma \\ k \end{array}\right)\left(\begin{array}{c} k\\ s \end{array}\right){\left(-1\right)}^{k}\frac{\alpha^{\gamma }\delta^{2\gamma +s}{\left(2+\delta \right)}^{\gamma -m}}{{\left(1+\delta \right)}^{2\gamma +2s}}\text{.}$

Using integration, we get the final expression

$$I_{R}\left(\gamma \right)=\frac{1}{1-\gamma }\log\left(\sum_{m=0}^{\infty }\sum_{k=0}^{\infty }\sum_{s=0}^{\infty }W_{mks}\;\frac{\left(m+s\right)!}{{\left(k+\gamma \right)}^{m+s+1}\delta^{m+s+1}}\right)$$

### 3.5 Mean residual life

The extra lifetime that is expected for the survival of an object of interest is termed mean residual life.

(6) $$\mu \left(t\right)=\frac{1}{S\left(t\right)}\int_{t}^{\infty }xg\left(x\right)dx-t\;for\;t\;>\;0\text{.}$$

Considering

$$\int_{t}^{\infty }xg\left(x\right)dx=\sum_{k=0}^{\infty }\sum_{s=0}^{\infty }\frac{\alpha \delta^{s+2}{\left(-1\right)}^{k}\left(\begin{array}{c} \alpha -1\\ k \end{array}\right)\left(\begin{array}{c} k\\ s \end{array}\right)}{{\left(1+\delta \right)}^{2s+2}}\int_{t}^{\infty }x\left(\left(2+\delta \right)x^{s}+x^{s+1}\right)e^{-\left(k+1\right)\delta x}dx\text{.}$$

Take an integral part and make a transformation $\left(k+1\right)\delta x=y\;and\;x=\frac{y}{\left(k+1\right)\delta }$.

$$\int_{t}^{\infty }xg\left(x\right)dx=\int_{t}^{\infty }x\left(\left(2+\delta \right)x^{s}+x^{s+1}\right)e^{-\left(k+1\right)\delta x}dx$$

$$=\left(2+\delta \right)\int_{\left(k+1\right)\delta t}^{\infty }{\left(\frac{y}{\left(k+1\right)\delta }\right)}^{s+1}e^{-y}\frac{dy}{\left(k+1\right)\delta }+\int_{\left(k+1\right)\delta t}^{\infty }{\left(\frac{y}{\left(k+1\right)\delta }\right)}^{s+2}e^{-y}\frac{dy}{\left(k+1\right)\delta }=\frac{1}{{\left(k+1\right)}^{s+2}\delta^{s+2}}\left[\left(2+\delta \right)\int_{\left(k+1\right)\delta t}^{\infty }y^{s+1}e^{-y}dy+\frac{1}{\left(k+1\right)\delta }\int_{\left(k+1\right)\delta t}^{\infty }y^{s+2}e^{-y}dy\right]$$

$$by\;using\;\int_{a}^{+\infty }z^{k}e^{-z}dz=\Gamma \left(k+1,a\right)$$

$$=\frac{1}{{\left(k+1\right)}^{s+2}\delta^{s+2}}\left[\left(2+\delta \right)\Gamma (2+s,\left(k+1)\delta t\right)+\frac{\Gamma \left(3+s,\left(k+1\right)\delta t\right)}{\left(k+1\right)\delta }\right]$$

$$=\frac{\delta \left(2+\delta \right)\left(k+1\right)\Gamma (2+s,\left(k+1)\delta t\right)+\Gamma \left(3+s,\left(k+1\right)\delta t\right)}{{\left(k+1\right)}^{s+3}\delta^{s+3}}$$

Substituting in equation (6)

$$\int_{t}^{\infty }xg\left(x\right)dx=\sum_{k=0}^{\infty }\sum_{s=0}^{\infty }\frac{\alpha \delta^{s+2}{\left(-1\right)}^{k}\left(\begin{array}{c} \alpha -1\\ k \end{array}\right)\left(\begin{array}{c} k\\ s \end{array}\right)}{{\left(1+\delta \right)}^{2s+2}}\left[\frac{\delta \left(2+\delta \right)\left(k+1\right)\Gamma (2+s,\left(k+1)\delta t\right)+\Gamma \left(3+s,\left(k+1\right)\delta t\right)}{{\left(k+1\right)}^{s+3}\delta^{s+3}}\right]$$

and

$$\mu \left(t\right)=\frac{1}{S\left(t\right)}\sum_{k=0}^{\infty }\sum_{s=0}^{\infty }\frac{\alpha \delta^{s+2}{\left(-1\right)}^{k}\left(\begin{array}{c} \alpha -1\\ k \end{array}\right)\left(\begin{array}{c} k\\ s \end{array}\right)}{{\left(1+\delta \right)}^{2s+2}}\frac{\delta \left(2+\delta \right)\left(k+1\right)\Gamma \left(2+s,\left(k+1\right)\delta t\right)+\Gamma \left(3+s,\left(k+1\right)\delta t\right)}{{\left(k+1\right)}^{s+3}\delta^{s+3}}-t\text{.}$$

### 3.6 Stress-strength reliability (SSR)

Suppose $X\;∼EXL\left(\delta ,\alpha_{1}\right)and\;Y∼EXL\left(\delta ,\alpha_{2}\right)$ then SSR parameter R can be obtained as

$$R=P\left(X>Y\right)=\int_{0}^{\infty }P\left(X>Y/Y=y\right)f_{Y}\left(y\right)dy$$

$$=\int_{0}^{\infty }\left[1-{\left[1-\left(1+\frac{\delta y}{{\left(1+\delta \right)}^{2}}\right)e^{-\delta y}\right]}^{\alpha_{1}}\right]\frac{\alpha_{2}\delta^{2}\left(2+\delta +y\right)e^{-\delta y}}{{\left(1+\delta \right)}^{2}}{\left[1-\left(1+\frac{\delta y}{{\left(1+\delta \right)}^{2}}\right)e^{-\delta y}\right]}^{\alpha_{2}-1}dy$$

$$=1-\int_{0}^{\infty }\frac{\alpha_{2}\delta^{2}\left(2+\delta +y\right)e^{-\delta y}}{{\left(1+\delta \right)}^{2}}{\left[1-\left(1+\frac{\delta y}{{\left(1+\delta \right)}^{2}}\right)e^{-\delta y}\right]}^{\alpha_{1}+\alpha_{2}-1}dy$$

Now by multiplying with $\alpha_{1}+\alpha_{2}$, we get

$$=1-\int_{0}^{\infty }\frac{\alpha_{2}\left(\alpha_{1}+\alpha_{2}\right)\delta^{2}\left(2+\delta +y\right)e^{-\delta y}}{\left(\alpha_{1}+\alpha_{2}\right){\left(1+\delta \right)}^{2}}{\left[1-\left(1+\frac{\delta y}{{\left(1+\delta \right)}^{2}}\right)e^{-\delta y}\right]}^{\alpha_{1}+\alpha_{2}-1}dy$$

$$=1-\frac{\alpha_{2}}{\alpha_{1}+\alpha_{2}}\int_{0}^{\infty }\frac{\left(\alpha_{1}+\alpha_{2}\right)\delta^{2}\left(2+\delta +y\right)e^{-\delta y}}{{\left(1+\delta \right)}^{2}}{\left[1-\left(1+\frac{\delta y}{{\left(1+\delta \right)}^{2}}\right)e^{-\delta y}\right]}^{\alpha_{1}+\alpha_{2}-1}dy$$

$$=\frac{\alpha_{1}}{\alpha_{1}+\alpha_{2}}\text{.}$$

## 4 Parameter estimation

In this section, we utilize six methods to estimate the parameters of the EXL distribution. A complete simulation analysis was also carried out to discover the most effective estimating approach.

### 4.1 Maximum likelihood estimation

Let $x_{1},x_{2},x_{3},\ldots ,x_{n}$ be a random sample of size n taken from the EXL distribution. The log-likelihood function is given by

$$L\left(\alpha ,\delta \right)=n\;\ln\left(\alpha \right)+2n\;\ln\left(\delta \right)-\delta \sum_{i=1}^{n}x_{i}-2nln\left(1+\delta \right)+\sum_{i=1}^{n}\ln\left(2+\delta +x_{i}\right)+\left(\alpha -1\right)\sum_{i=1}^{n}\ln\left[1-\left(1+\frac{\delta x_{i}}{{\left(1+\delta \right)}^{2}}\right)e^{-\delta x_{i}}\right]\text{.}$$

For MLE, the above equation will be maximized for both parameters

$$\frac{\partial L\left(\alpha ,\delta \right)}{\partial \delta }=\frac{2n}{\delta }-\sum_{i=1}^{n}x_{i}-\frac{2n}{1+\delta }+\sum_{i=1}^{n}\frac{1}{2+\delta +x_{i}}+\left(\alpha -1\right)\sum_{i=1}^{n}\frac{\left[\left(\frac{2\delta x_{i}}{{\left(1+\delta \right)}^{3}}-\frac{x_{i}}{{\left(1+\delta \right)}^{2}}\right)+x_{i}\left(1+\frac{\delta x_{i}}{{\left(1+\delta \right)}^{2}}\right)\right]e^{-\delta x_{i}}}{1-\left(1+\frac{\delta x_{i}}{{\left(1+\delta \right)}^{2}}\right)e^{-\delta x_{i}}}\text{,}$$

and

$$\frac{\partial L\left(\alpha ,\delta \right)}{\partial \alpha }=\frac{n}{\alpha }+\sum_{i=1}^{n}\ln\left[1-e^{-\delta x_{i}}\left(1+\frac{\delta x_{i}}{{\left(1+\delta \right)}^{2}}\right)\right]\text{.}$$

### 4.2 Maximum product spacing estimation

This estimation approach was proposed by Ref. [25] as an alternative to ML estimation. The geometric mean of the differences may be maximized to get the MPSE of parameters.

$$G\left(\alpha ,\delta \right)={\left[\prod_{i=1}^{n+1}D_{i}\left(\alpha ,\delta \right)\right]}^{\frac{1}{n+1}}\text{,}$$

or by minimizing the log of the geometric mean of sample spacing given by

$$H\left(\alpha ,\delta \right)=\frac{1}{n+1}\sum_{i=1}^{n+1}\log\;D_{i}\left(\alpha ,\delta \right)$$

### 4.3 Anderson-Darling estimation

The ADE of EXL distribution can be obtained by minimizing the following statistic

$$A=-n-\frac{1}{n}\sum_{i=1}^{n}\left(2i-1\right)\left\{\log\left[G\left(x_{i}\right)\right]+\log\left[1-G\left(x_{n+1-i}\right)\right]\right\}\text{,}$$

and

$$A\left(\alpha ,\delta \right)=-n-\frac{1}{n}\sum_{i=1}^{n}\left(2i-1\right)\left\{\log\left[{\left(1-\left(1+\frac{\delta x_{i}}{{\left(1+\delta \right)}^{2}}\right)e^{-\delta x_{i}}\right)}^{\alpha }\right]+\log\left[1-{\left(1-\left(1+\frac{\delta x_{n+1-i}}{{\left(1+\delta \right)}^{2}}\right)e^{-\delta x_{n+1-i}}\right)}^{\alpha }\right]\right\}\text{.}$$

### 4.4 Cramer von misses estimation

The CVME of EXL distribution can be obtained by minimizing the following distance

$$\text{CVME}=\frac{1}{12n}+\sum_{i=1}^{n}{\left(G\left(x_{i}\right)-\frac{2i-1}{2n}\right)}^{2}\text{,}$$

and

$$\text{CVME}\left(\alpha ,\delta \right)=\frac{1}{12n}+\sum_{i=1}^{n}{\left({\left[1-\left(1+\frac{\delta x_{i}}{{\left(1+\delta \right)}^{2}}\right)e^{-\delta x_{i}}\right]}^{\alpha }-\frac{2i-1}{2n}\right)}^{2}\text{.}$$

### 4.5 Ordinary and weighted least square estimation

The ordinary least squares (OLSE) of the EXL distribution can be obtained by minimizing the distance between theoretical and empirical cdf. The OLSEs are obtained by minimizing the following function

$$S\left(\alpha ,\delta \right)=\sum_{i=1}^{n}{\left[G\left(x_{i}|\alpha ,\delta \right)-\frac{i}{n+1}\right]}^{2}$$

and

$$S\left(\alpha ,\delta \right)=\sum_{i=1}^{n}{\left\{{\left[1-\left(1+\frac{\delta x_{i}}{{\left(1+\delta \right)}^{2}}\right)e^{-\delta x_{i}}\right]}^{\alpha }-\frac{i}{n+1}\right\}}^{2}$$

The Weighted Least Square Estimators (WLSE) can be obtained by minimizing the following distance

$$W\left(\alpha ,\delta \right)=\sum_{i=1}^{n}\left[\frac{{\left(n+1\right)}^{2}.\left(n+2\right)}{\left(n+1-i\right)i}\right]{\left[G\left(x_{i}|\alpha ,\delta \right)-\frac{i}{n+1}\right]}^{2}$$

and

$$W\left(\alpha ,\delta \right)=\sum_{i=1}^{n}\left[\frac{{\left(n+1\right)}^{2}.\left(n+2\right)}{\left(n+1-i\right)i}\right]{\left[{\left[1-\left(1+\frac{\delta x_{i}}{{\left(1+\delta \right)}^{2}}\right)e^{-\delta x_{i}}\right]}^{\alpha }-\frac{i}{n+1}\right]}^{2}$$

## *5* Simulation study

In the following section, we conduct a simulation study to evaluate the efficiency of EXL estimators. A random sample is generated from the EXL distribution with some selected values of parameters. The following algorithm is used for generating samples from the new model:

i. Generation of n values of u from a uniform distribution with parameters (0, 1)
ii. Computation of n values of Xu (random numbers of EXLD) using the relation

$$x_{u}=-\frac{{\left(1+\delta \right)}^{2}}{\delta }-\frac{1}{\delta }W_{-1}\left[\frac{{\left(1+\delta \right)}^{2}}{e^{{\left(1+\delta \right)}^{2}}}\left(u^{\frac{1}{\alpha }}-1\right)\right]$$

iii. The number of replications is taken as 10000.
iv. The different parameter values are taken as
(α,δ) = (0.5, 0.25), (0.5, 2.0), (1.0, 0.25), (1.0, 2.0), (2.0, 0.25), (2.0, 1.0).
v. The sample sizes used for the study are taken n = 20, 50, 100, and 300. The selected sample sizes reflect small, moderate, and large samples respectively.
vi. The performance of estimators was evaluated through the bias, mean relative error (MRE), and mean square errors (MSE). The Bias, MRE, and MSE were calculated from 10,000 samples of each selected sample size.

The simulation results are given in Table 2, Table 3, Table 4, Table 5, Table 6, Table 7.

**Table 2:** Estimation of EXLD parameters for =${\left(\alpha =0.50,\delta =0.25\right)}^{T}$.

n	Para.	Est.	MLE	ADE	CVME	OLSE	WLSE	MPSE
20	α	AE	0.57582	0.53845	0.59600	0.52406	0.52861	0.46550
		AB	0.07582	0.03845	0.09600	0.02406	0.02861	0.03451
		MRE	0.15163	0.07690	0.19201	0.04811	0.05721	0.06901
		MSE	0.04521	0.03772	0.09071	0.05411	0.04840	0.02395
	δ	AE	0.28184	0.26375	0.28570	0.25362	0.25713	0.22886
		AB	0.03184	0.01375	0.03570	0.00362	0.00713	0.02114
		MRE	0.12735	0.05501	0.14278	0.01447	0.02851	0.08454
		MSE	0.00822	0.00718	0.01260	0.00893	0.00817	0.00562
50	α	AE	0.52407	0.51156	0.52972	0.50526	0.50958	0.47179
		AB	0.02407	0.01156	0.02972	0.00526	0.00958	0.02821
		MRE	0.04814	0.02313	0.05945	0.01053	0.01915	0.05642
		MSE	0.01013	0.01034	0.01609	0.01325	0.01123	0.00815
	δ	AE	0.26103	0.25455	0.26260	0.25060	0.25326	0.23488
		AB	0.01103	0.00455	0.01260	0.00060	0.00326	0.01512
		MRE	0.04411	0.01819	0.05040	0.00240	0.01303	0.06047
		MSE	0.00232	0.00240	0.00345	0.00301	0.00258	0.00209
100	α	AE	0.51370	0.50751	0.51602	0.50419	0.50753	0.48325
		AB	0.01370	0.00751	0.01602	0.00419	0.00753	0.01675
		MRE	0.02741	0.01503	0.03203	0.00837	0.01505	0.03350
		MSE	0.00461	0.00489	0.00664	0.00599	0.00511	0.00408
	δ	AE	0.25582	0.25268	0.25670	0.25080	0.25267	0.24061
		AB	0.00582	0.00268	0.00670	0.00080	0.00267	0.00939
		MRE	0.02328	0.01073	0.02678	0.00318	0.01066	0.03757
		MSE	0.00105	0.00113	0.00152	0.00141	0.00118	0.00101
300	α	AE	0.50464	0.50263	0.50543	0.50159	0.50306	0.49218
		AB	0.00464	0.00263	0.00543	0.00159	0.00306	0.00782
		MRE	0.00928	0.00527	0.01086	0.00317	0.00613	0.01564
		MSE	0.00137	0.00152	0.00195	0.00188	0.00155	0.00133
	δ	AE	0.25208	0.25104	0.25236	0.25042	0.25125	0.24584
		AB	0.00208	0.00104	0.00236	0.00042	0.00125	0.00416
		MRE	0.00832	0.00417	0.00944	0.00167	0.00498	0.01663
		MSE	0.00032	0.00036	0.00047	0.00046	0.00037	0.00032

**Table 3:** Estimation of EXLD parameters for =${\left(\alpha =0.50,\delta =2.00\right)}^{T}$.

n	Para.	Est.	MLE	ADE	CVME	OLSE	WLSE	MPSE
20	α	AE	0.56513	0.53149	0.58179	0.51910	0.52372	0.46642
		AB	0.06513	0.03149	0.08179	0.01910	0.02372	0.03358
		MRE	0.13026	0.06298	0.16358	0.03821	0.04743	0.06715
		MSE	0.03667	0.03144	0.07508	0.04516	0.03996	0.02095
	δ	AE	2.33260	2.15679	2.40450	2.08438	2.11278	1.82891
		AB	0.33260	0.15679	0.40450	0.08438	0.11278	0.17109
		MRE	0.16630	0.07840	0.20225	0.04219	0.05639	0.08554
		MSE	0.84726	0.72276	1.51413	0.99728	0.90647	0.49571
50	α	AE	0.52465	0.51189	0.52681	0.50512	0.50998	0.47702
		AB	0.02465	0.01189	0.02681	0.00512	0.00998	0.02298
		MRE	0.04930	0.02378	0.05362	0.01024	0.01995	0.04596
		MSE	0.00929	0.00922	0.01336	0.01120	0.00978	0.00739
	δ	AE	2.12476	2.05788	2.13522	2.01977	2.04665	1.88002
		AB	0.12476	0.05788	0.13522	0.01977	0.04665	0.11998
		MRE	0.06238	0.02894	0.06761	0.00988	0.02332	0.05999
		MSE	0.21549	0.22096	0.33155	0.27824	0.24015	0.17286
100	α	AE	0.51045	0.50501	0.51262	0.50214	0.50510	0.48284
		AB	0.01045	0.00501	0.01262	0.00214	0.00510	0.01716
		MRE	0.02090	0.01001	0.02523	0.00428	0.01019	0.03431
		MSE	0.00403	0.00431	0.00571	0.00524	0.00449	0.00372
	δ	AE	2.05884	2.03065	2.07152	2.01503	2.03048	1.91630
		AB	0.05884	0.03065	0.07152	0.01503	0.03048	0.08370
		MRE	0.02942	0.01533	0.03576	0.00752	0.01524	0.04185
		MSE	0.09372	0.10362	0.14459	0.13162	0.10908	0.08569
300	α	AE	0.50344	0.50171	0.50412	0.50071	0.50207	0.49203
		AB	0.00344	0.00171	0.00412	0.00071	0.00207	0.00797
		MRE	0.00688	0.00341	0.00825	0.00142	0.00414	0.01595
		MSE	0.00122	0.00135	0.00169	0.00164	0.00137	0.00121
	δ	AE	2.01949	2.00978	2.02227	2.00385	2.01158	1.96080
		AB	0.01949	0.00978	0.02227	0.00385	0.01158	0.03920
		MRE	0.00974	0.00489	0.01113	0.00192	0.00579	0.01960
		MSE	0.02879	0.03265	0.04221	0.04092	0.03316	0.02837

**Table 4:** Estimation of EXLD parameters for =${\left(\alpha =1.00,\delta =0.25\right)}^{T}$.

n	Para.	Est.	MLE	ADE	CVME	OLSE	WLSE	MPSE
20	α	AE	1.19087	1.09640	1.27278	1.07592	1.08320	0.91633
		AB	0.19087	0.09640	0.27278	0.07592	0.08320	0.08367
		MRE	0.19087	0.09640	0.27278	0.07592	0.08320	0.08367
		MSE	0.28083	0.22231	1.02547	0.46828	0.37334	0.12776
	δ	AE	0.27306	0.25801	0.27562	0.24972	0.25288	0.22906
		AB	0.02306	0.00801	0.02562	0.00028	0.00288	0.02094
		MRE	0.09224	0.03205	0.10247	0.00110	0.01151	0.08378
		MSE	0.00474	0.00426	0.00748	0.00560	0.00497	0.00365
50	α	AE	1.06466	1.03237	1.07825	1.01660	1.02778	0.93641
		AB	0.06466	0.03237	0.07825	0.01660	0.02778	0.06359
		MRE	0.06466	0.03237	0.07825	0.01660	0.02778	0.06359
		MSE	0.05927	0.05882	0.09521	0.07535	0.06461	0.04372
	δ	AE	0.25848	0.25275	0.25900	0.24915	0.25171	0.23651
		AB	0.00848	0.00275	0.00900	0.00085	0.00171	0.01349
		MRE	0.03392	0.01100	0.03598	0.00339	0.00684	0.05396
		MSE	0.00155	0.00161	0.00225	0.00202	0.00173	0.00147
100	α	AE	1.03098	1.01609	1.03836	1.00892	1.01649	0.95714
		AB	0.03098	0.01609	0.03836	0.00892	0.01649	0.04286
		MRE	0.03098	0.01609	0.03836	0.00892	0.01649	0.04286
		MSE	0.02447	0.02625	0.03792	0.03366	0.02781	0.02131
	δ	AE	0.25438	0.25159	0.25474	0.24988	0.25159	0.24156
		AB	0.00438	0.00159	0.00474	0.00012	0.00159	0.00844
		MRE	0.01753	0.00638	0.01896	0.00049	0.00635	0.03374
		MSE	0.00073	0.00079	0.00104	0.00098	0.00082	0.00073
300	α	AE	1.01025	1.00489	1.01112	1.00163	1.00583	0.97996
		AB	0.01025	0.00489	0.01112	0.00163	0.00583	0.02004
		MRE	0.01025	0.00489	0.01112	0.00163	0.00583	0.02004
		MSE	0.00733	0.00829	0.01096	0.01055	0.00848	0.00707
	δ	AE	0.25141	0.25039	0.25128	0.24968	0.25055	0.24614
		AB	0.00141	0.00039	0.00128	0.00032	0.00055	0.00387
		MRE	0.00563	0.00155	0.00512	0.00129	0.00219	0.01546
		MSE	0.00023	0.00025	0.00032	0.00032	0.00026	0.00023

**Table 5:** Estimation of EXLD parameters for =${\left(\alpha =1.00,\delta =2.00\right)}^{T}$.

n	Para.	Est.	MLE	ADE	CVME	OLSE	WLSE	MPSE
20	α	AE	1.16411	1.08246	1.21466	1.05272	1.06343	0.92239
		AB	0.16411	0.08246	0.21466	0.05272	0.06343	0.07761
		MRE	0.16411	0.08246	0.21466	0.05272	0.06343	0.07761
		MSE	0.19576	0.16159	0.43376	0.24059	0.22210	0.09800
	δ	AE	2.23435	2.09859	2.26163	2.02251	2.05003	1.83627
		AB	0.23435	0.09859	0.26163	0.02251	0.05003	0.16373
		MRE	0.11717	0.04930	0.13082	0.01125	0.02502	0.08187
		MSE	0.44371	0.38501	0.67363	0.47666	0.43500	0.30030
50	α	AE	1.06065	1.03388	1.07536	1.02061	1.02992	0.94557
		AB	0.06065	0.03388	0.07536	0.02061	0.02992	0.05443
		MRE	0.06065	0.03388	0.07536	0.02061	0.02992	0.05443
		MSE	0.04873	0.04970	0.07982	0.06394	0.05422	0.03655
	δ	AE	2.08318	2.03507	2.09629	2.00657	2.02698	1.88707
		AB	0.08318	0.03507	0.09629	0.00657	0.02698	0.11293
		MRE	0.04159	0.01754	0.04814	0.00329	0.01349	0.05647
		MSE	0.12896	0.13403	0.19531	0.16967	0.14535	0.11479
100	α	AE	1.02670	1.01277	1.03181	1.00586	1.01298	0.96079
		AB	0.02670	0.01277	0.03181	0.00586	0.01298	0.03922
		MRE	0.02670	0.01277	0.03181	0.00586	0.01298	0.03922
		MSE	0.01995	0.02129	0.02993	0.02690	0.02246	0.01783
	δ	AE	2.04019	2.01474	2.04404	2.00006	2.01476	1.92614
		AB	0.04019	0.01474	0.04404	0.00006	0.01476	0.07386
		MRE	0.02009	0.00737	0.02202	0.00003	0.00738	0.03693
		MSE	0.05828	0.06293	0.08500	0.07930	0.06584	0.05655
300	α	AE	1.01121	1.00705	1.01331	1.00486	1.00786	0.98406
		AB	0.01121	0.00705	0.01331	0.00486	0.00786	0.01594
		MRE	0.01121	0.00705	0.01331	0.00486	0.00786	0.01594
		MSE	0.00629	0.00695	0.00902	0.00866	0.00708	0.00602
	δ	AE	2.01549	2.00759	2.01717	2.00268	2.00892	1.96855
		AB	0.01549	0.00759	0.01717	0.00268	0.00892	0.03145
		MRE	0.00774	0.00380	0.00859	0.00134	0.00446	0.01573
		MSE	0.01841	0.02053	0.02623	0.02555	0.02091	0.01840

**Table 6:** Estimation of EXLD parameters for =${\left(\alpha =2.00,\delta =0.25\right)}^{T}$.

n	Para.	Est.	MLE	ADE	CVME	OLSE	WLSE	MPSE
20	α	AE	2.53533	2.28850	3.00522	2.36326	2.29014	1.83500
		AB	0.53533	0.28850	1.00522	0.36326	0.29014	0.16500
		MRE	0.26766	0.14425	0.50261	0.18163	0.14507	0.08250
		MSE	2.26132	1.72434	344.75185	78.52130	3.39842	0.83453
	δ	AE	0.26923	0.25565	0.27068	0.24819	0.25113	0.23057
		AB	0.01923	0.00565	0.02068	0.00181	0.00113	0.01943
		MRE	0.07692	0.02260	0.08270	0.00724	0.00451	0.07772
		MSE	0.00369	0.00333	0.00566	0.00440	0.00395	0.00300
50	α	AE	2.17578	2.09366	2.21477	2.05979	2.08533	1.86149
		AB	0.17578	0.09366	0.21477	0.05979	0.08533	0.13852
		MRE	0.08789	0.04683	0.10739	0.02990	0.04266	0.06926
		MSE	0.35216	0.33867	0.61922	0.46680	0.38773	0.23570
	δ	AE	0.25759	0.25234	0.25757	0.24898	0.25144	0.23817
		AB	0.00759	0.00234	0.00757	0.00102	0.00144	0.01184
		MRE	0.03036	0.00937	0.03029	0.00409	0.00574	0.04734
		MSE	0.00118	0.00121	0.00168	0.00152	0.00130	0.00113
100	α	AE	2.08239	2.04424	2.10014	2.02752	2.04522	1.90249
		AB	0.08239	0.04424	0.10014	0.02752	0.04522	0.09751
		MRE	0.04120	0.02212	0.05007	0.01376	0.02261	0.04876
		MSE	0.13726	0.14684	0.22189	0.19271	0.15712	0.11355
	δ	AE	0.25345	0.25088	0.25353	0.24928	0.25085	0.24210
		AB	0.00345	0.00088	0.00353	0.00072	0.00085	0.00791
		MRE	0.01380	0.00352	0.01411	0.00288	0.00341	0.03162
		MSE	0.00055	0.00059	0.00077	0.00073	0.00061	0.00056
300	α	AE	2.02865	2.01490	2.03106	2.00781	2.01719	1.95465
		AB	0.02865	0.01490	0.03106	0.00781	0.01719	0.04535
		MRE	0.01433	0.00745	0.01553	0.00391	0.00860	0.02268
		MSE	0.04075	0.04581	0.06125	0.05847	0.04682	0.03837
	δ	AE	0.25142	0.25049	0.25130	0.24990	0.25063	0.24669
		AB	0.00142	0.00049	0.00130	0.00010	0.00063	0.00331
		MRE	0.00566	0.00197	0.00521	0.00041	0.00253	0.01325
		MSE	0.00018	0.00020	0.00025	0.00025	0.00020	0.00018

**Table 7:** Estimation of EXLD parameters for =${\left(\alpha =2.00,\delta =1.00\right)}^{T}$.

n	Para.	Est.	MLE	ADE	CVME	OLSE	WLSE	MPSE
20	α	AE	2.47332	2.24784	2.71086	2.22711	2.23261	1.83539
		AB	0.47332	0.24784	0.71086	0.22711	0.23261	0.16461
		MRE	0.23666	0.12392	0.35543	0.11356	0.11631	0.08231
		MSE	2.02864	1.50916	16.65339	6.98238	4.89143	0.78308
	δ	AE	1.08375	1.02909	1.09309	0.99895	1.01002	0.92372
		AB	0.08375	0.02909	0.09309	0.00105	0.01002	0.07628
		MRE	0.08375	0.02909	0.09309	0.00105	0.01002	0.07628
		MSE	0.06537	0.05790	0.10012	0.07544	0.06841	0.05060
50	α	AE	2.16662	2.09588	2.21286	2.06897	2.08932	1.87515
		AB	0.16662	0.09588	0.21286	0.06897	0.08932	0.12485
		MRE	0.08331	0.04794	0.10643	0.03449	0.04466	0.06243
		MSE	0.31088	0.30803	0.57324	0.43662	0.35661	0.21183
	δ	AE	1.03394	1.01345	1.03659	1.00095	1.01005	0.95414
		AB	0.03394	0.01345	0.03659	0.00095	0.01005	0.04586
		MRE	0.03394	0.01345	0.03659	0.00095	0.01005	0.04586
		MSE	0.02068	0.02119	0.02991	0.02658	0.02276	0.01919
100	α	AE	2.07916	2.04360	2.09223	2.02533	2.04365	1.91232
		AB	0.07916	0.04360	0.09223	0.02533	0.04365	0.08768
		MRE	0.03958	0.02180	0.04612	0.01266	0.02182	0.04384
		MSE	0.12194	0.12937	0.19015	0.16644	0.13714	0.10159
	δ	AE	1.01821	1.00779	1.01848	1.00093	1.00755	0.97153
		AB	0.01821	0.00779	0.01848	0.00093	0.00755	0.02848
		MRE	0.01821	0.00779	0.01848	0.00093	0.00755	0.02848
		MSE	0.00954	0.01026	0.01351	0.01271	0.01069	0.00933
300	α	AE	2.02423	2.01361	2.02984	2.00832	2.01583	1.95600
		AB	0.02423	0.01361	0.02984	0.00832	0.01583	0.04400
		MRE	0.01212	0.00680	0.01492	0.00416	0.00792	0.02200
		MSE	0.03497	0.03952	0.05348	0.05112	0.04048	0.03344
	δ	AE	1.00495	1.00189	1.00573	0.99994	1.00251	0.98571
		AB	0.00495	0.00189	0.00573	0.00006	0.00251	0.01429
		MRE	0.00495	0.00189	0.00573	0.00006	0.00251	0.01429
		MSE	0.00300	0.00336	0.00428	0.00419	0.00341	0.00309

It is evident from the above simulation tables that absolute bias, mean relative error, and MSE reduce with the upsurge in sample size for all estimation methods. For small sample size (n = 20) ADE and MPSE methods show better results regarding bias, MSE, and MSE. While for large samples MLE method performs better than others.

## 6 Application of EXL distribution

The distribution's use is proven in the manuscript using three real datasets. For comparison purposes following probability distributions are utilized such as exponential (Exp), Lindley (L), XLindley (XL), generalized Lindley (GL), Weibull, power Lindley (PL), and Nadarajah–Haghighi (NH). The MLE method is used to estimate the parameters of models.

Several measures are available in the literature that are used for the selection of fitted distribution. In this study, we will consider six imperative measures: Akaike Information Criteria (AIC), Bayesian Information Criteria (BIC), Anderson Darling (AD), Cramer Von Mises (CVM), and Kolmogorov Smirnov (KS), respectively.

**Fig. 3:**
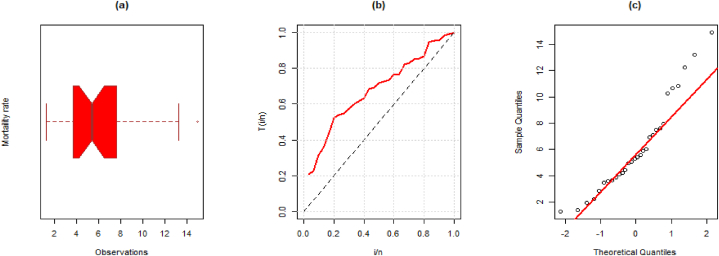
(a) Boxplot, (b) TTT plot, and (c) Q-Q plot for the first data.

**Data I:** The first dataset is related to the mortality rate due to COVID-19 for 30 days (31st March to April 30, 2020) and was recorded for the Netherlands. This data was discussed by Ref. [26]. The observations are; 14.918, 10.656, 12.274, 10.289, 10.832, 7.099, 5.928, 13.211, 7.968, 7.584, 5.555, 6.027, 4.097, 3.611, 4.960, 7.498, 6.940, 5.307, 5.048, 2.857, 2.254, 5.431, 4.462, 3.883, 3.461, 3.647, 1.974, 1.273, 1.416 and 4.235. Some basic descriptive measures corresponding to the first dataset are mean = 6.1565, variance = 12.4843, median = 5.369, minimum = 1.273, maximum = 14.918, skewness = 0.8340, and kurtosis = 2.9534. Further, the visual representation such as the boxplot, TTT plot, and Q-Q plot for the first dataset is presented in Fig. 3 (a)-(c).

**Fig. 4:**
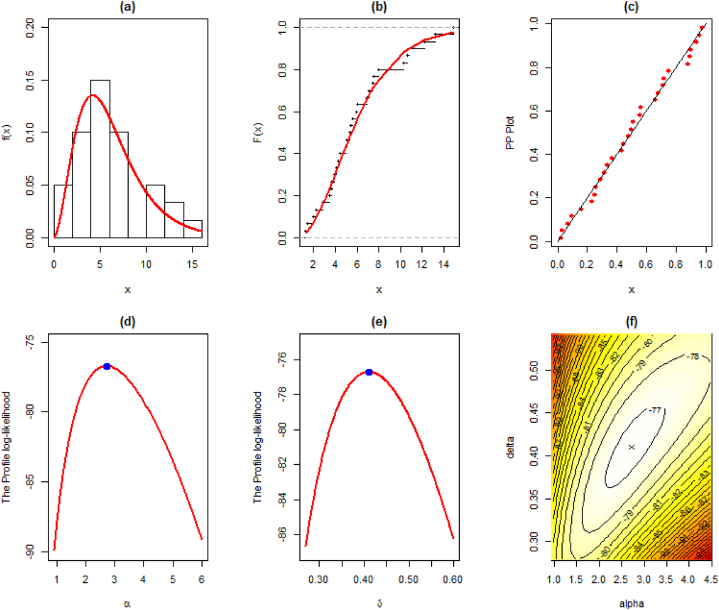
Illustration of the fitted (a) pdf, (b) cdf, (c) PP, (d)–(e) profile log-likelihood, and (f) contour plots of the EXL model for the first data.

The MLEs, SE, and goodness-of-fit measures for this dataset are given in Table 8. We also provide the visual comparison using fitted pdf, cdf, PP, profile log-likelihood, and contour plots given in Fig. 4 (a)-(f).

**Table 8:** Maximum Likelihood Estimate and Goodness of Fit measures for the first data.

Distribution	Parameter		Log-Lik.	AIC	BIC	KS	
	Estimate	S.E.				Statistic	Sig.
EXP	0.1625	0.0297	−84.525	171.050	172.452	0.263	0.025
LD	0.2885	0.0377	−79.964	161.928	163.329	0.179	0.257
XLD	0.2631	0.0347	−81.261	164.523	165.924	0.201	0.152
GLD	0.4180	0.0678	−76.732	157.464	160.267	0.080	0.981
	2.2660	0.7058					
Weibull	0.0260	0.0153	−77.034	158.069	160.871	0.100	0.896
	1.8801	0.2590					
PL	1.3774	0.1648	−77.009	158.019	160.821	0.092	0.942
	0.1421	0.0494					
NH	30.353	20.443	−79.913	163.826	166.628	0.193	0.190
	0.0035	0.0023					
EXLD	2.7254	0.8215	−76.713	157.426	160.229	0.080	0.982
	0.4103	0.0649

**Fig. 5:**
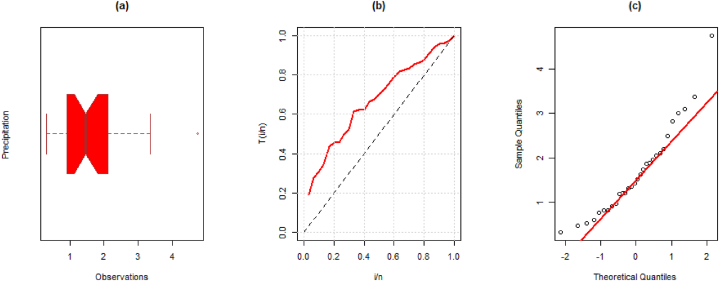
(a) Boxplot, (b) TTT plot, and (c) Q-Q plot for the second data.

**Data II:** The second data given below is regarding precipitation in inches. The data was utilized and discussed by Ref. [27]. The data observations are; 0.77, 1.74, 0.81, 1.20, 1.95, 1.20, 0.47, 1.43, 3.37, 2.20, 3.00, 3.09, 1.51, 2.10, 0.52, 1.62, 1.31, 0.32, 0.59, 0.81, 2.81, 1.87, 1.18, 1.35, 4.75, 2.48, 0.96, 1.89, 0.90 and 2.05. Some computations of this dataset are mean = 1.670, variance = 1.0012, median = 1.470, skewness = 1.0867, and kurtosis = 4.2069. Further, the visual representation such as the boxplot, TTT plot, and Q-Q plot for the first dataset is presented in Fig. 5 (a)-(c).

**Fig. 6:**
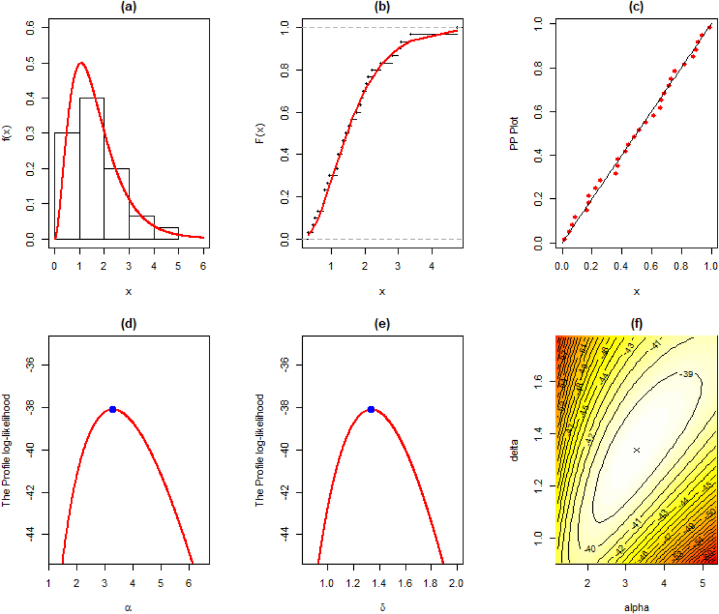
Illustration of the fitted (a) pdf, (b) cdf, (c) PP, (d)–(e) profile log-likelihood, and (f) contour plots of the EXL model for the second data.

The MLEs, SE, and goodness-of-fit measures for this dataset are given in Table 9. We also provide the visual comparison using fitted pdf, cdf, PP (probability-probability), profile log-likelihood, and contour plots given in Fig. 6 (a)-(f).

**Table 9:** Maximum Likelihood Estimate and Goodness of Fit measures for the second data.

Distribution	Parameter		Log-Lik.	AIC	BIC	KS	
	Estimate	S.E.				Statistic	Sig.
EXP	0.5970	0.1090	−45.474	92.949	94.350	0.2352	0.072
LD	0.9097	0.1247	−43.144	88.287	89.689	0.1883	0.238
XLD	0.7799	0.1108	−44.548	91.906	92.497	0.2142	0.128
GLD	1.4528	0.2327	−38.120	80.240	83.043	0.0872	0.999
	2.8188	0.8830					
Weibull	0.3154	0.0906	−38.643	81.287	84.089	0.0689	0.999
	1.8090	0.2491					
PL	1.5263	0.1924	−38.872	81.745	84.548	0.0682	0.999
	0.6460	0.1243					
NH	22.071	29.338	−41.428	86.856	89.659	0.1579	0.442
	0.0175	0.0239					
EXLD	3.2795	1.0301	−38.088	80.175	82.978	0.0626	1.000
	1.3370	0.2144

**Fig. 7:**
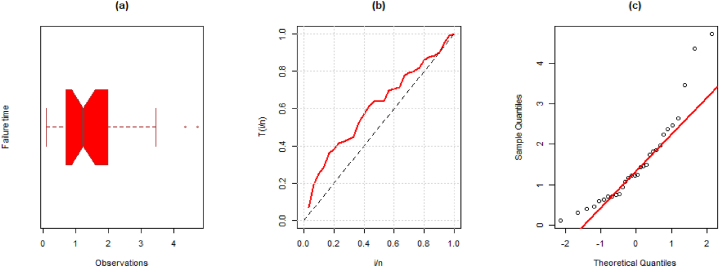
(a) Boxplot, (b) TTT, and (c) Q-Q plot for the third data.

**Data III:** The third dataset, which has been examined by Ref. [28], corresponds to the period between failures for 30 repairable goods. The data observations are; 1.43, 0.11, 0.71, 0.77, 2.63, 1.49, 3.46, 2.46, 0.59, 0.74, 1.23, 0.94, 4.36, 0.40, 1.74, 4.73, 2.23, 0.45, 0.70, 1.06, 1.46, 0.30, 1.82, 2.37, 0.63, 1.23, 1.24, 1.97, 1.86 and 1.17. Some numerical values of this dataset are mean = 1.5427, variance = 1.2717, minimum = 0.1100, maximum = 4.7300, skewness = 1.2955, and kurtosis = 4.3192. Further, the visual representation such as the boxplot, TTT plot, and Q-Q plot for the first dataset is presented in Fig. 7 (a)-(c).

**Fig. 8:**
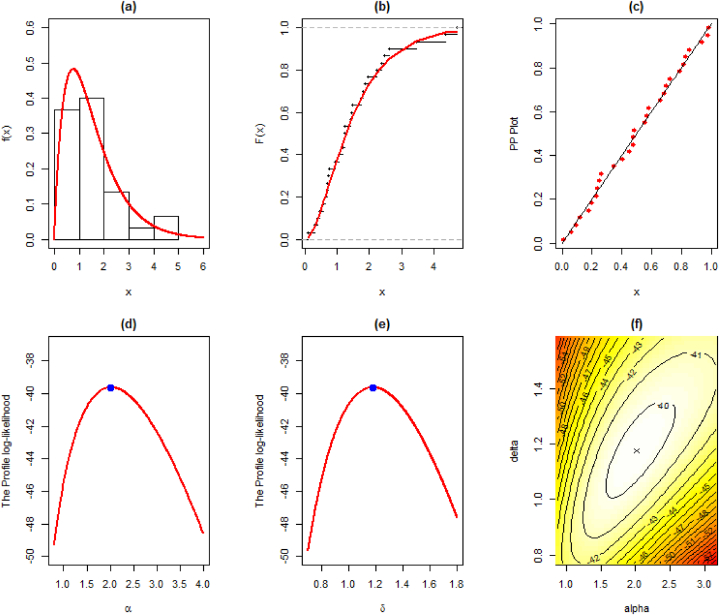
Illustration of the fitted (a) pdf, (b) cdf, (c) PP, (d)–(e) profile log-likelihood, and (f) contour plots of the EXL model for the third data.

The MLEs, SE, and goodness-of-fit measures for this dataset are given in Table 10. We also provide the visual comparison using fitted pdf, cdf, PP, profile log-likelihood, and contour plots given in Fig. 8 (a)-(f).

**Table 10:** Maximum Likelihood Estimate and Goodness of Fit measures for the third data.

Distribution	Parameter		Log-Lik.	AIC	BIC	KS	
	Estimate	S.E.				Statistic	Sig.
EXP	0.6482	0.1183	−43.005	88.010	89.412	0.1844	0.259
LD	0.9762	0.1345	−41.547	85.095	86.496	0.1407	0.593
XLD	0.8367	0.1197	−42.435	86.870	88.272	0.1669	0.373
GLD	1.2868	0.2218	−39.657	83.315	86.117	0.0724	0.998
	1.7688	0.5020					
Weibull	0.4560	0.1141	−39.910	83.820	86.623	0.0748	0.996
	1.4634	0.2029					
PL	1.2700	0.1647	−40.090	84.181	86.984	0.0781	0.993
	0.8255	0.1445					
NH	4.4429	6.5150	−41.153	86.307	89.110	0.1131	0.838
	0.0978	0.1640					
EXLD	2.0174	0.5717	−39.618	83.236	86.038	0.0675	0.999
	1.1766	0.2024

Six estimate methods have been used to achieve one of the main goals of this study, which is to get the best estimators for three data sets. The different estimators for data sets based on various estimating methods are listed in Table 11.

**Table 11:** Estimation and Goodness for all three datasets.

Data	Method↓Statistics→		δ	α	KS	*P*-value
Mortality Rate	MLE		0.4103	2.7241	0.0800	0.9824
	ADE		1.1777	2.0077	0.0654	0.9995
	CVME		1.2102	2.0946	0.0677	0.9991
	OLSE		1.1429	1.8909	0.0578	1.0000
	WLSE		1.1217	1.8402	0.0590	0.9999
	MPSE		1.0313	1.6127	0.0728	0.9973
Precipitation	MLE		1.3371	3.2797	0.0626	0.9998
	ADE		1.2909	3.0476	0.0655	0.9995
	CVME		1.2854	3.0371	0.0639	0.9997
	OLSE		1.2177	2.7095	0.0701	0.9985
	WLSE		1.2150	2.6848	0.0720	0.9977
	MPSE		1.1880	2.5813	0.0720	0.9977
Failure Times	MLE		1.1767	2.0173	0.0674	0.9992
	ADE		1.1777	2.0077	0.0654	0.9995
	CVME		1.2102	2.0946	0.0677	0.9991
	OLSE		1.1429	1.8909	0.0578	1.0000
	WLSE		1.1217	1.8402	0.0590	0.9999
	MPSE		1.0313	1.6127	0.0728	0.9973

It should be noted that all considered estimation approaches analyze failure rate, precipitation, and mortality rate data effectively. However, the OLSE method is best for failure rate and mortality rate data, while the ADE method is best for precipitation data.

## 7 Bayesian analysis

The estimation of parameters using the Bayesian approach is discussed in this section. A prior distribution is a requirement for each parameter to estimate the parameter using the Bayesian approach. Thus, gamma distribution is assumed for both parameters δ and α considering these parameters are real and positive numbers. The joint prior distribution can be written as:

$$\pi \left(\delta ,\alpha \right)\propto \delta^{a_{1}-1}e^{-\delta b_{1}}\alpha^{a_{2}-1}e^{-\alpha b_{2}}$$

Where, $a_{1}$, $a_{2}$, $b_{1}$ and $b_{2}$ are the known hyperparameters.

For the generation of data from the joint prior distribution, the MCMC method is implemented using MCMCpack available in R software. We generated 1005000 samples for each parameter. The first 5000 samples were considered as a burn-in period, which is usually used for minimization of effects for initial values. The Bayes estimates can be obtained as the mean of the posterior distribution. The 95 % highest posterior density (HPD) intervals for parameters are obtained from the posterior distribution of parameters. Trace plots and Geweke diagnostics were used to monitor the convergence of simulated sequences.

The results for estimates, standard deviation, 95 % HPD intervals, and Geweke's Z-score are presented in Table 12.

**Table 12:** Bayesian estimates, SE, HPD, and Geweke's score for both datasets.

Data		Estimates	SD	95 % HPD interval	Geweke's Z-score
Mortality Rate	$\hat{\delta }$	0.1975	0.0368	(0.1264, 0.2691)	0.8362
	$\hat{\alpha }$	0.6142	0.1265	(0.3852, 0.8736)	0.2746
Precipitation	$\hat{\delta }$	0.4319	0.0897	(0.2564, 0.6034)	−0.2584
	$\hat{\alpha }$	0.5399	0.1123	(0.3404, 0.7736)	−0.0279
Failure Times	$\hat{\delta }$	0.4308	0.0909	(0.2560, 0.6101)	−0.2164
	$\hat{\alpha }$	0.4980	0.1028	(0.3162, 0.7146)	−0.0899

**Fig. 9:**
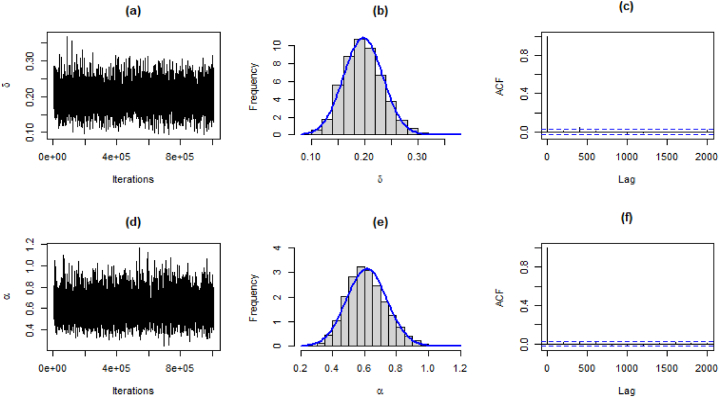
Traceplot, Posterior densities, and ACF plot for the first data.

**Fig. 10:**
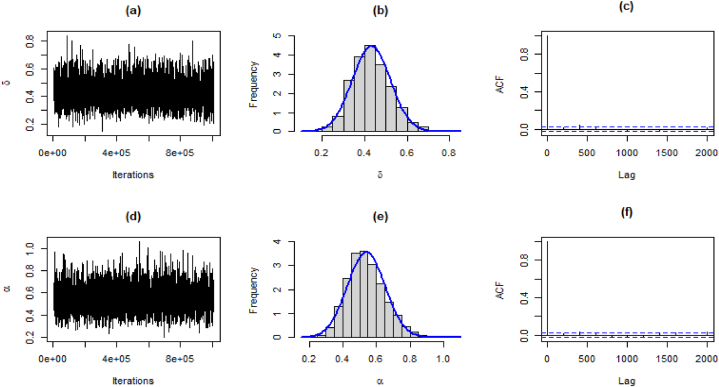
Traceplot, Posterior densities, and ACF plot for the second data.

**Fig. 11:**
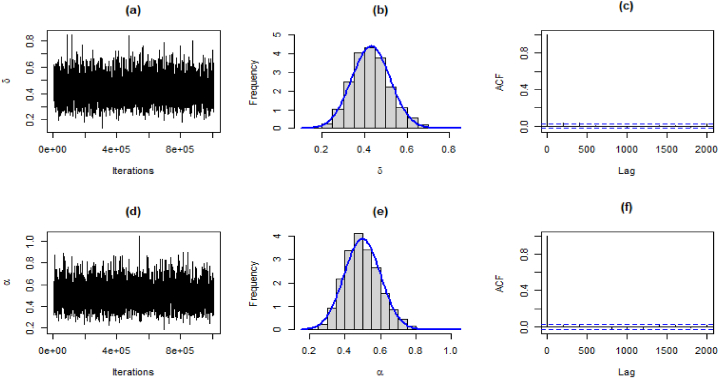
Traceplot, Posterior densities, and ACF plot for the third data.

The traceplot, and histogram of posterior density are used for the evaluation of the MCMC iterations. The posterior samples for the parameters for the first dataset are shown in Fig. 9(a)-(f), Fig. 10(a)-(f), and Fig. 11(a)-(f), respectively.

## 8 Conclusion

In this study, the exponentiated XLindley distribution is proposed and studied. A new shape parameter is introduced to enhance the flexibility of the XLindley distribution. There are three subfamilies of distribution which based on the new parameter α (α=1, α<1, and α>1). The proposed distribution has exponentially decreased behavior for α<1, while for α>1 the distribution is unimodal, positively skewed with many variations at the start, and becomes flattered as the value of the second parameter δ decreases. The mean and variance decrease with the increase in parameter α. The model parameters were estimated using six different estimation methods. A comprehensive simulation was carried out for various combinations of parameters and different sample sizes. Estimates improve as the sample size increases. The Bayesian technique with MCMC was utilized for estimation parameters. Traceplots and Geweke diagnostics were used to monitor the convergence of simulated sequences. The application of EXL distribution is illustrated by three datasets from different fields such as mortality rate due to COVID-19, precipitation, and failure time of repairable items. The proposed EXL distribution is compared with the existing seven-lifetime models: exponential, Lindley, XLindley, generalized Lindley, Weibull, power Lindley, and Nadarajah-–Haghighi. For all the datasets, the goodness of fit measures and graphical presentations are evident that EXL distribution outperformed all mentioned models by acquiring minimum values of the goodness of fit criteria.

Future research on the new three-parameter distribution may focus on a variety of different topics. Here are a few examples:

• Additional examination of the proposed distribution can be explored in different dimensions. One of the most preferable directions is to propose its neutrosophic extension [[29], [30], [31], [32]] to analyze datasets with indeterminacy.
• Bayesian estimation can be used to estimate the model parameters using different loss functions and different approximation techniques.

## Delimitations and limitations

The proposed model will be applicable in such situations where the conditions of the model will be satisfied. Unlike the existing XLindley distribution, the newly proposed model will apply to various kinds of data sets. This model will apply to lifetime data ranging from 0 to ∞.

## Data availability statement

Data included in article/supplementary material/referenced in article.

## CRediT authorship contribution statement

**Abdullah M. Alomair:** Writing – review & editing, Supervision, Funding acquisition. **Mukhtar Ahmed:** Writing – original draft. **Saadia Tariq:** Supervision. **Muhammad Ahsan-ul-Haq:** Writing – review & editing, Writing – original draft, Visualization, Methodology, Formal analysis. **Junaid Talib:** Methodology.

## Declaration of competing interest

The Authors have no conflict of interest.

## Appendix

Proof of quantile function:

From the cdf of EXLD. The equation $u=G_{X}\left(x\right)$ will be solved for x.

$${\left[1-\left(1+\frac{\delta x}{{\left(1+\delta \right)}^{2}}\right)e^{-\delta x}\right]}^{\alpha }=u$$

$$\left[{\left(1+\delta \right)}^{2}+\delta x\right]e^{-\delta x}=\left(1-u^{\frac{1}{\alpha }}\right){\left(1+\delta \right)}^{2}$$

Multiply both sides of the above equation by $e^{-{\left(1+\delta \right)}^{2}.}$.

$$\left[{\left(1+\delta \right)}^{2}+\delta x\right]e^{-\delta x}e^{-{\left(1+\delta \right)}^{2}}=\left(1-u^{\frac{1}{\alpha }}\right){\left(1+\delta \right)}^{2}e^{-{\left(1+\delta \right)}^{2}}$$

$$-\left[{\left(1+\delta \right)}^{2}+\delta x\right]e^{-\left[{\left(1+\delta \right)}^{2}+\delta x\right]}=\left(u^{\frac{1}{\alpha }}-1\right){\left(1+\delta \right)}^{2}e^{-{\left(1+\delta \right)}^{2}}$$

Applying the Lambert W function on both sides we get

$$W\left[-\left[{\left(1+\delta \right)}^{2}+\delta x\right]e^{-\left[{\left(1+\delta \right)}^{2}+\delta x\right]}\right]=W\left[\left(u^{\frac{1}{\alpha }}-1\right){\left(1+\delta \right)}^{2}e^{-{\left(1+\delta \right)}^{2}}\right]$$

As we know that $W\left[ze^{z}\right]=z$, then the equation becomes

$$-\left[{\left(1+\delta \right)}^{2}+\delta x\right]=W\left[\left(u^{\frac{1}{\alpha }}-1\right){\left(1+\delta \right)}^{2}e^{-{\left(1+\delta \right)}^{2}}\right]$$

For $\alpha >0,\delta >0\;and\;x>0,{\left(1+\delta \right)}^{2}+\delta x>0$ and it is also checked $\left(u^{\frac{1}{\alpha }}-1\right){\left(1+\delta \right)}^{2}e^{-{\left(1+\delta \right)}^{2}}\in \left(\frac{1}{e},0\right)$ since 0 < u < 1. Thus, by using the properties of the negative branch $W_{-1}$ of the Lambert W function. Hence

$$x_{u}=-\frac{{\left(1+\delta \right)}^{2}}{\delta }-\frac{1}{\delta }W_{-1}\left[\frac{{\left(1+\delta \right)}^{2}}{e^{{\left(1+\delta \right)}^{2}}}\left(u^{\frac{1}{\alpha }}-1\right)\right]\text{.}$$
